# ADAP Promotes Degranulation and Migration of NK Cells Primed During *in vivo Listeria monocytogenes* Infection in Mice

**DOI:** 10.3389/fimmu.2019.03144

**Published:** 2020-01-22

**Authors:** Martha A. L. Böning, Stephanie Trittel, Peggy Riese, Marco van Ham, Maxi Heyner, Martin Voss, Gerald P. Parzmair, Frank Klawonn, Andreas Jeron, Carlos A. Guzman, Lothar Jänsch, Burkhart Schraven, Annegret Reinhold, Dunja Bruder

**Affiliations:** ^1^Infection Immunology Group, Institute of Medical Microbiology, Infection Control and Prevention, Health Campus Immunology, Infectiology and Inflammation, Otto-von-Guericke University Magdeburg, Magdeburg, Germany; ^2^Institute of Molecular and Clinical Immunology, Health Campus Immunology, Infectiology and Inflammation, Otto-von-Guericke University Magdeburg, Magdeburg, Germany; ^3^Immune Regulation Group, Helmholtz Centre for Infection Research, Braunschweig, Germany; ^4^Vaccinology and Applied Microbiology, Helmholtz Centre for Infection Research, Braunschweig, Germany; ^5^Cellular Proteome Research, Helmholtz Centre for Infection Research, Braunschweig, Germany

**Keywords:** ADAP, natural killer cells, cytotoxicity, *Listeria monocytogenes*, *in vivo* infection, migration, IL-10, CD11a

## Abstract

The adhesion and degranulation-promoting adaptor protein (ADAP) serves as a multifunctional scaffold and is involved in the formation of immune signaling complexes. To date only limited and moreover conflicting data exist regarding the role of ADAP in NK cells. To extend existing knowledge we investigated ADAP-dependency of NK cells in the context of *in vivo* infection with the intracellular pathogen *Listeria monocytogenes* (*Lm*). *Ex vivo* analysis of infection-primed NK cells revealed impaired cytotoxic capacity in NK cells lacking ADAP as indicated by reduced CD107a surface expression and inefficient perforin production. However, ADAP-deficiency had no global effect on NK cell morphology or intracellular distribution of CD107a-containing vesicles. Proteomic definition of ADAPko and wild type NK cells did not uncover obvious differences in protein composition during the steady state and moreover, similar early response patterns were induced in NK cells upon infection independent of the genotype. In line with protein network analyses that suggested an altered migration phenotype in naïve ADAPko NK cells, *in vitro* migration assays uncovered significantly reduced migration of both naïve as well as infection-primed ADAPko NK cells compared to wild type NK cells. Notably, this migration defect was associated with a significantly reduced expression of the integrin CD11a on the surface of splenic ADAP-deficient NK cells 1 day post-*Lm* infection. We propose that ADAP-dependent alterations in integrin expression might account at least in part for the fact that during *in vivo* infection significantly lower numbers of ADAPko NK cells accumulate in the spleen i.e., the site of infection. In conclusion, we show here that during systemic *Lm* infection in mice ADAP is essential for efficient cytotoxic capacity and migration of NK cells.

## Introduction

The coupling of transmembrane receptors to intracellular signaling pathways is mediated by adapter proteins that are made up of various protein domains without enzymatic or transcriptional activity. Adaptor proteins are central players involved in a number of cellular processes including cell proliferation, migration, and cell cycle regulation (1). The Adhesion and degranulation-promoting adapter protein ADAP, also known as Fyn-binding protein (Fyb) or SLAP-130 serves, amongst others, as a scaffold adapter protein specific for the hematopoietic lineage that so far has been mainly studied in the context of the activation of and effector functions in T cells (2). ADAP consists of several domains that can associate with proteins involved in cell migration, cellular adhesion and re-arrangement of the cytoskeleton in T cells (3). Moreover, ADAP plays an important role in T cell receptor- and chemokine receptor-mediated activation of integrins (inside-out signaling) and mediates signals derived from the interaction of integrins on T cells with ligands on target cells (outside-in signaling) (4, 5). ADAP-deficient T cells show reduced migration toward chemokines (6), impaired formation of the immunological synapse (4, 5) and impaired activation, differentiation and resident memory formation during acute infections (6, 7).

Next to its well-documented expression in T cells, ADAP is also expressed in other cells of the hematopoietic lineage including platelets, myeloid cells (8, 9) unconventional T cells, such as NKT, CD8α, and TCRγδ T cells (10) as well as in Natural Killer (NK) cells (11–13). NK cells are large granular lymphocytes which play a crucial role in the innate immune responses toward tumors and intracellular pathogens (14, 15). NK cells are able to identify malignant and infected cells and their activation occurs following integration of signals delivered by multiple activating and inhibitory receptors expressed on their surface (16, 17). In case the activating signals predominate, target cell death is induced by delivery of the cytolytic effector molecules perforin and granzymes (18). In addition to their inherent cytotoxic function, NK cells exhibit the capacity to produce effector cytokines and chemokines (19, 20), with IFN-γ being the principal NK cell cytokine produced early on during infections (21, 22). IFN-γ plays a central role for the activation of other immune cells needed for effective immunity to pathogens (23, 24). Thus, NK cells act as important immune regulators during infection and inflammation (25). In order to effectively fulfill their immunological functions, numerous cytokines, such as Interleukin (IL)-15, IL-18 (26, 27), IL-2, IL-4, IL-21 (27), and type I interferons (28) secreted by other immune cells are needed to prime NK cell activation, proliferation, and their differentiation into fully armed effector cells (29, 30).

NK cells develop from the same common lymphoid precursor as CD8^+^ T cells (31) and both cell types share hallmark features like cytotoxicity and effector cytokine secretion. In light of their common ancestry, the fundamental role of ADAP in T cells and the usage of similar signaling pathways in T cells and NK cells, Fostel et al. proposed a similar requirement for ADAP in NK cell development and function. In direct contrast to their expectation, loss of ADAP in NK cells did neither affect NK cell development and function, nor cytotoxicity, IFN-γ production, anti-tumor response, and LFA-1-dependent conjugate formation with target cells (13). Several years later another study came to the opposite conclusion that NK cell activation would rely on ADAP. Here the authors demonstrated both impaired IFN-γ production and cytotoxicity of NK cells lacking ADAP (32). Only shortly later, Rajasekaran et al. uncovered a striking and ADAP-dependent uncoupling of cytokine secretion and cytotoxicity in NK cells (12). Using an experimental model system of CD137 and NKG2D induced *in vitro* stimulation of NK cells in combination with comprehensive analysis of the molecular signaling machinery utilized during NK cell activation the authors convincingly demonstrated that upon stimulation ADAP connects to the CBM signalosome consisting of the proteins Carma1, Bcl-10, and MALT1. This ADAP-CBM complex was essential for the production of cytokines and chemokines but not for cytotoxicity in murine and human NK cells (12). Taken together, available data regarding the role of ADAP in NK cells are conflicting ranging from a crucial role of ADAP in both pro-inflammatory cytokine production and cytotoxicity (32), an ADAP-dependent uncoupling of cytokine production and cytotoxicity with only cytokine production being dependent on ADAP (12) and the finding that ADAP in NK cells is fully dispensable for their function (13).

While the above mentioned studies utilized different experimental settings and NK cell stimulating ligands they all have in common that ADAP-dependency of principal NK cell functions was largely characterized either in primary or IL-2-activated NK cells following *in vitro* stimulation of selected NK cell activating receptors. To our knowledge no published data exist regarding the involvement of ADAP in NK cell functions *in vivo*. Given the fact that under physiological conditions NK cell activation is not induced by the stimulation of a single receptor but is the consequence of integration of signals derived by multiple activating and inhibitory receptors, NK cell activation *in vivo* is likely more complex than activation induced under *in vitro* conditions. Thus, we sought to extend existing knowledge regarding the role of ADAP in NK cell function to an *in vivo* setting. *Lm* is a facultative intracellular pathogen causing systemic infections that is frequently used as model pathogen. *In vivo* infection of mice with *Lm* is known to induce effective NK cell activation (33, 34) in a process involving a complex cellular network of macrophages, neutrophils, and dendritic cells as well as a plethora of soluble mediators derived from these cells (35, 36). Thus, *Lm* infection, that is well-established in our laboratory (6), represents a suitable tool to study the role of ADAP in NK cell priming, cytokine production and cytotoxicity during *in vivo* infection.

## Materials and Methods

### Mice

ADAP wild type and knock out (ADAPko) mice have been described before (37) and were bred in the animal facility at the Helmholtz Center for Infection Research in Braunschweig (Germany) or in the animal facility of the Medical Faculty of the Otto-von-Guericke University Magdeburg (Germany). Mice were kept under specific pathogen-free conditions in environmentally-controlled clean rooms and were used at 8–24 weeks of age. If not otherwise stated, experiments were performed using male mice and were approved by the local government agencies (Niedersächsisches Landesamt für Verbraucherschutz und Lebensmittelsicherheit and Landesamt für Verbraucherschutz, Sachsen-Anhalt). Mice containing the knockout first allele C57BL/6N-Fyb^tm1a(EUCOMM)Hmgu^/Cnrm (38) were sourced from the EUCOMM project and were purchased from the European Mouse Mutant Archive EMMA. In this so called “knockout-first strategy” the ADAP allele is modified up- and downstream of its critical exon 2, the largest exon of ADAP. The lacZ and neomycin-resistance cassettes were both removed by breeding with transgenic mice expressing a Flp recombinase resulting in floxed alleles (containing *loxP* sites) and restoring the wild type (9). To generate conditional knockout mice with deletion of ADAP in the NK cell lineage, mice with floxed alleles were crossed with NKp46-iCre knock-in mice kindly provided by Prof. Eric Vivier (39). The presence or absence of the *FRT* sites, the *loxP* sites, the gene of interest, and the respective Cre transgene were checked routinely by PCR using genomic DNA isolated from ear tissue. To investigate specific effects of ADAP deletion and to exclude off-target effects of Cre recombinase, ADAP^wt/wt^ × Cre^het^, and ADAP^fl/fl^ × Cre^het^ were always used as littermates. NK cell maturation in the bone marrow as well as distribution of mature NK cells in spleen and peripheral blood was similar in both genotypes (40).

### *Listeria Monocytogenes* Infection

The *Listeria monocytogenes* (*Lm*) strain 10403S expressing ovalbumin was used for mouse infection experiments as described before (6, 41). Frozen stocks of *Lm* were thawed and subsequently diluted in brain heart infusion medium (BHI) followed by overnight growth on agar plates. One day before the infection, an overnight liquid culture (BHI) was prepared which was diluted with fresh medium (1:5) and cultivated at 37°C for 4 h before bacteria were harvested. Bacterial numbers were determined by measuring OD_600_ using a spectrophotometer and based on a previously established OD/cell-density correlation curve the suspension was further diluted in sterile PBS to the desired infection dose per mouse. Mice were infected by intravenous injection into the tail vein with the calculated dose of 2.5 × 10^4^ bacteria suspended in 100 μl of PBS. To determine the actual infectious dose serial dilutions were plated on BHI agar followed by an overnight incubation of the plate at 37°C. The colonies were counted on the next day which, corrected for the dilution factor, showed the actual number of bacteria that had been applied to the mice. Body weight loss (infection dose: 5 × 10^4^ bacteria) as an indicator for disease severity was measured daily over a period of 8 days. To determine colony forming units (CFU) in the organs of infected animals, mice were sacrificed at the indicated time points, spleens were collected and half of the spleens was homogenized in lysis buffer (PBS containing 0.2% IGEPAL CA-630). Ten microliters of 10-fold serial dilutions were plated on BHI agar plates. The bacterial count (CFU) was obtained by counting the colonies after incubation for 24 h at 37°C.

### Quantitative RT-PCR

RNA was isolated from splenocytes using the RNeasy Mini Kit (Qiagen, Hilden, Germany) according to the manufacturer's instructions. RNA was eluted in 100 μl nuclease-free water. RNA content was determined with the NanoDrop ND-1000 spectrophotometer (Thermo Fisher Scientific, Massachusetts, USA). For cDNA synthesis ~1 μg of RNA was transcribed using the Maxima First Strand cDNA Synthesis Kit for RT-qPCR according to the manufactures instructions (Thermo Fisher Scientific). cDNA was used as a template for real-time PCR using SYBR Green I (Roche). RPS9 was used as a housekeeping gene for normalization. Quantitative RT-PCRs were run in duplicates in the LightCycler 480 system II using the primers summarized in Table 1. mRNA sequences were derived from ncbi gene database and intron spanning real time PCR primers were designed accordingly using the web based assay design center (https://lifescience.roche.com/en_de/brands/universal-probe-library.html#assay-design-center) tool from Roche company. All primers were used in a final concentration of 500 nmol/l. The relative expression of the investigated target genes was calculated in relation to the housekeeping gene with the aid of the ΔΔCt method and the LightCycler480 software.

**Table 1:** Primers used for qRT-PCR.

**Gene symbol**	**Forward primer sequence (length)**	**Reverse primer sequence (length)**	**Annealing temp. (°C)**
*Ccl3*	TGCCCTTGCTGTTCTTCTCT(20)	GTGGAATCTTCCGGCTGTAG(20)	60
*Ccl4*	CCCCTCTCTCTCCTCTTGCT(20)	GAGGGTCAGAGCCCATTG(18)	60
*Ccl5*	TGCAGAGGACTCTGAGACAGC(21)	GAGTGGTGTCCGAGCCATA(19)	60
*Il-2*	CAAGCAGGCCACAGAATTGAAA(22)	GGCACTCAAATGTGTTGTCAGA(22)	58.4
*Il-12b*	GTAACCAGAAAGGTGCGTTCC(21)	GAACACATGCCCACTTGCTG(20)	59.8; 59.4
*Il-15*	GGTCCTCCTGCAAGTCTCTC(20)	GGTGGATTCTTTCCTGACCTCTC(23)	61.4; 62.4
*Il-18*	GAAAGCCGCCTCAAACCTTC(20)	CCAGGTCTCCATTTTCTTCAGG(22)	59.4; 60.3
*Il-21*	ATCTTCTTGGGGACAGTGGC(20)	AGTGCCCCTTTACATCTTGTGG(22)	59.4; 60.3
*Rps9*	CTGGACGAGGGCAAGATGAAGC(22)	TGACGTTGGCGGATGAGCACA(21)	58.0

### Serum Preparation and Cytokine Analyses

Mice were sacrificed and blood was obtained by puncture of the heart. Samples were incubated for 30 min at room temperature and for another 30 min at 4°C. After short centrifugation (14,000 rpm) the supernatant was collected and stored at −20°C. For the detection of IL-1α, IL-1β, IL-2, IL-4, IL-10, IL-12p40, IL-12p70, IL-15, IL-18, IL-21, IFN-γ, TNF-α and IFN-β in serum samples a flow cytometry based custom multiplex detection assay (custom LegendPlex, mouse, BioLegend) was used according to the manufacturer's recommendations.

### Proteome Analyzes

FACS-sorted NK cells (NKp46^+^) were lysed in a buffer containing 1% SDS, 1× Complete protease inhibitor cocktail (Roche), 50 mM HEPES pH 8.5, 10 mM DTT for 5 min at 95°C, and rested for 5 min on ice. Benzonase was added and samples were incubated at 37°C for 30 min. After incubation, 5 mM tris(2-carboxyethyl)phosphine (TCEP) was added for 30 min, and 10 mM methyl methanethiosulfonate (MMTS) for 5 min, to reduce and protect cysteine residues, respectively. Protein purification, protein digestion and peptide purification was performed according to a slightly adapted Single-Pot Solid-Phase-enhanced Sample Preparation (SP3) protocol (42, 43). Sequencing grade trypsin (Promega, Fitchburg, WI, USA) was added at a ratio of 1:20 weight per weight in 50 mM HEPES pH 8. After < 14 h incubation at 37°C, samples were slightly acidified using formic acid (FA) shaken and incubated overnight at RT after raising the acetonitrile concentration to at least 95%. Beads containing the adsorbed peptides were washed once with pure acetonitrile and were dried by air. Peptides were eluted in a first step with 20 μl 2% DMSO for 30 min, and in a second step with 20 μl 0.065% FA, 500 mM KCl in 30% acetonitrile (ACN) for 30 min. Peptides were vacuum dried and dissolved in 0.2% trifluoroacetic acid/3% ACN for subsequent ultracentrifugation (50,000× g, 30 min, RT). LC-MS/MS analyses of purified and desalted peptides were performed on a Dionex UltiMate 3000 n-RSLC system connected to an Orbitrap Fusion™ Tribrid™ mass spectrometer (Thermo Scientific, Waltham, MA, USA). Peptides of each sample were loaded onto a C18 precolumn (3 μm RP18 beads, Acclaim, 0.075 × 20 mm), washed for 3 min at a flow rate of 6 μl/min and separated on a C18 analytical column (3 mm, Acclaim PepMap RSLC, 0.075 mm × 50 cm, Dionex, Sunnyvale, CA, USA) at a flow rate of 200 nl/min via a linear 120 min gradient from 97% MS buffer A (0.1% FA) to 25% MS buffer B (0.1% FA, 80% ACN), followed by a 30 min gradient from 25% MS buffer B to 62% MS buffer B. The LC system was operated with the Chromeleon software (version 6.8, Dionex) embedded in the Xcalibur software suite (version 3.0.63, Thermo Scientific). The effluent was electro-sprayed by a stainless steel emitter (Thermo Scientific). Using the Xcalibur software, the mass spectrometer was controlled and operated in the “top speed” mode, allowing the automatic selection of as many doubly and triply charged peptides in a 3 s time window as possible, and the subsequent fragmentation of these peptides. Peptide fragmentation was carried out using the higher energy collisional dissociation mode and peptides were measured in the ion trap (HCD/IT). MS/MS raw data files were processed via the Proteome Discoverer program (version 2.3, Thermo Scientific) using Mascot (version 2.4.1, Matrix Science) as search machine and fasta files from the Swiss-Prot/UniProt database from January 2018. Used Mascot search parameters were: maximum missed cleavage site: 1, precursor mass tolerance: 10 ppm, fragment mass tolerance: 0.05 Da. Oxidation of methionine was set as a variable modification and modification of cysteine by MMTS was set as fixed modification. Filters used in Proteome Discoverer were peptide confidence: high, search engine rank: 1, and false discovery rate: 1%. The entire mass spectrometry proteomics data have been deposited to the ProteomeXchange Consortium via the PRIDE partner repository with the data set identifier PXD016305.

### Microscopy

FACS sorted NK cells were seeded onto poly-L-lysine (Sigma) coated coverslips and allowed to adhere for 30 min. Cells were fixed by using 4% paraformaldehyde (Sigma) in PBS for 20 min followed by washing with PBS. Cells were permeabilized using 0.15% Triton-X100 (Sigma) in PBS for 5 min followed by blocking using 1% BSA (Sigma) in PBS containing 0.05% Tween-20 (Roth) for 1 h. Antibodies (anti-α-tubulin: ab52866/Abcam; FITC-labeled anti-CD107a: clone 1D4B/BioLegend; Alexa594-labeled anti-rabbit: A11037/Invitrogen) were applied in blocking solution and staining was performed for 2 h at RT. Cells were washed three times with PBS containing 0.05% Tween-20 followed by dehydration using first 70% and then 100% ethanol. Samples were dried by air and mounted using Mowiol (Roth). Light microscopy was carried out on an inverted microscope (ECLIPSE Ti-E; Nikon) with standard epifluorescence illumination (Intensilight C-HGFIE; Nikon) and 100×/NA1.4 plan-apochromatic objective. Images were acquired with a back-illuminated, cooled charge-coupled-device camera (DS2-Qi2; Nikon) driven by NIS-Elements (Nikon). Data acquisition was performed in NIS-Elements.

### Flow Cytometry Analyzes

Spleens were flushed by heart perfusion with 10 ml PBS. Afterwards the spleens were meshed trough a cell strainer (100 μm) with a syringe plunger and splenocytes were pelleted by centrifugation (1,200 rpm, 10 min, 4°C). Erythrocyte lysis was performed with different concentration of sodium chloride (PBS containing 0.2 or 1.6% NaCl, respectively) and stopped with PBS. After centrifugation (see above) splenocytes were filtered through a cell strainer (30 μm), centrifuged and the single cell suspension was collected in IMDM-complete medium containing 10% FCS, 1% Penicillin/Streptavidin, 0.1% Gentamycin, and 0.1% 2-Mercaptoethanol. For flow cytometric analysis single cell suspensions were treated with anti-CD16/32 (BioLegend) to reduce unspecific antibody binding. For live/dead discrimination the Fixable Viability Dye (eFlour780, eBioscience) was used followed by staining with the following antibodies: anti-CD3ε (FITC, 145-2C11), anti-CD8a (FITC, 53-6.7), anti-Ly6C (PerCP-Cy5.5, HK1.4), anti-Ly6G (BV510, 1A8), anti-NK1.1 (PE-Cy7, PK136), anti-CD11b (BV421, M1/70), anti-CD49b (APC, DX5) (all Biolegend) as well as anti-CD27 (PE, LG.3A10, eBioscience). Afterwards, cells were fixed with 2% paraformaldehyde and analyzed with a BD FACSCanto II (BD Biosciences). Flow cytometric data were analyzed using the BD FACS Diva v6.1.3 software.

### Integrin Expression on NK Cells

Spleens were harvested from *Lm* infected or naïve ADAP^wt/wt^ × NKp46-Cre^het^ and conditional ADAP^fl/fl^ × NKp46-Cre^het^ mice and processed into single cell suspension. For flow cytometric analysis single cell suspensions were treated with anti-CD16/32 (BioLegend). Cell suspensions were furthermore surface stained with a lineage mix (anti-CD3e, 145-2C11; anti-CD4, GK1.5; anti-CD8, 53-6.7; anti-CD19, 6D5; and anti-TER-119, TER-119; all FITC from BioLegend, except anti-TER-119 which was from Thermo Fisher). Furthermore, the following antibodies were used: anti-NK1.1 (APC-Cy7, PK136), anti-CD122 (PE-Cy7, TM-ß1), anti-CD11b (BV510, M1/70), anti-CD18 (AF647, M18/2), anti-CXCR4 (APC, L276F12), anti-CD29 (PE, HMß1-1) all from BioLegend as well as anti-NKp46 (V450, 29A1.4, BD Biosciences), and anti-CD11a (PE, 2D7, BD Pharmingen). Afterwards, cells were analyzed with a BD LSR Fortessa (BD Biosciences). Flow cytometric data were analyzed using the BD FACS Diva v6.1.3 software.

### IL-10 Expression on NK Cells

Livers were harvested from *Lm* infected or naïve ADAP^wt/wt^ × NKp46-Cre^het^ and conditional ADAP^fl/fl^ × NKp46-Cre^het^ mice and cells were isolated using the mouse liver dissociation Kit (Miltenyi Biotech) and the gentleMACS dissociator device (Miltenyi Biotech) according to the manufactures instructions and processed into single cell suspension. Afterwards, each cell suspension was stimulated for 4 h at 37°C with phorbol myristate acetate (20 ng/ml, Sigma-Aldrich) and ionomycin (1 μg/ml, Sigma-Aldrich) in medium (IMDM-complete). After 1 h brefeldin A (1,000×, BioLegend) and Monensin (1,000×, BioLegend) were added. For flow cytometric analysis single cell suspensions were treated with anti-CD16/32 (BioLegend) after 4 h of stimulation. For live/dead discrimination the Fixable Viability Dye (eFlour780, eBioscience) was used followed by staining with the following antibodies: anti-B220 (FITC, RA3-6B2, BioLegend), anti-CD4 (FITC, RM4-5, BioLegend), anti-CD8 (FITC, 53-6.7, BioLegend), anti-CD3 (BV510, 17A2, BioLegend), anti-NK1.1 (PE, PK136, BD Pharmingen), anti-NKp46 (eFlour660, 29A1.4, Invitrogen). Cells were fixed with the Fixation/Permeabilization Kit (Thermo Fisher Scientific, Massachusetts, USA) and followed by intracellular staining for anti-IL-10 (BV421, JES5-16E3, BioLegend) for 30 min at 4°C. Cells were than analyzed using an Attune NxT Flow Cytometer (Thermo Fisher Scientific).

### NK Cell Isolation

Spleens were harvested from *Lm* infected or naïve mice and processed into single cell suspension without erythrocyte lyses. Untouched NK cells were isolated using the mouse NK cell Isolation Kit (Miltenyi Biotech) and the autoMACS device (Miltenyi Biotech) according to the manufactures instructions or were sorted using a BD FACSAria (BD Biosciences) cell sorter. The purity of autoMACS isolated NK cells was >86 and >88% after FACSAria cell sorting. For untouched cell sorting, NK cells were stained for live/dead discrimination with Fixable Viability Dye (eFlour780, eBioscience) followed by staining with the following antibodies: anti-CD3ε (FITC, 145-2C11), anti-Ly6G (BV510, A18), anti-CD11b (BV421, M1/70), anti-NK1.1 (Biotin, PK136), anti-CD49b (APC, DX5) (all BioLegend), and anti-CD27 (PE, LG.3A10, eBioscience) or with anti-CD3ε (APC, 145-2C11, BioLegend), anti-CD8a (PerCP-Cy5.5, 53-6.7, BioLegend), anti-CD4 (BV510, RM4-5, BioLegend), and anti-NK1.1 (PE, PK136, BD Bioscience). Biotinylated antibodies were counterstained with streptavidin (PE-Cy7, BioLegend).

### *In vitro* NK Cell Stimulation

Ninety-six-well U-bottom plates were coated overnight with anti-NK1.1 (1 μg/ml, Biotin, PK139, BioLegend) diluted in phosphate-buffered saline (PBS) at 4°C. Isolated NK cells (untouched) were added after removal of excessive anti-NK1.1 antibody. If indicated either IL-2 (3,000 Units/ml, BioLegend) and IL-12 (1 ng/ml, BioLegend) or phorbol myristate acetate (20 ng/ml, Sigma-Aldrich) and ionomycin (1 μg/ml, Sigma-Aldrich) were added to the wells and NK cells were stimulated for 4 h at 37°C. Controls were cultured in medium (IMDM-complete) without further stimulation. After 2 h brefeldin A (5 μg/ml, BioLegend) was added to all wells including the control wells. After 4 h *in vitro* stimulation NK cells were treated for live/dead discrimination with Fixable Viability Dye (eFlour780, eBioscience) and anti-CD16/32 (BioLegend) followed by staining with the following antibodies: anti-CD3ε (FITC, 145-2C11, BioLegend), anti-CD107a (Biotin, 1D4B, BioLegend), anti-CD49b (APC, DX5, BioLegend), anti-CD11b (PerCP-Cy5.5, M1/70, BioLegend). Cells were fixed with the Fixation/Permeabilization Kit (Thermo Fisher Scientific, Massachusetts, USA) followed by intracellular staining for anti-IFN-γ (BV421, XMG1.2, BioLegend) according to the manufacturer's instructions. Cells were than analyzed using an Attune NxT Flow Cytometer (Thermo Fisher Scientific). Biotinylated antibodies were counterstained with streptavidin (PE-Cy7, BioLegend).

### *In vitro* NK Cell Migration Assay

For *in vitro* migration assay a transwell system (Costar; Corning, USA) was used. Here, the NK cell attracting chemokine CXCL12 (250 ng/ml, R&D Systems) was added to the lower chamber in a total volume of 600 μl chemotaxis medium (RPMI containing 25 mM HEPES and 0.5% BSA). Total splenocytes obtained from untreated and *Lm* infected mice, respectively, were loaded to the upper chamber at a density of 5 × 10^5^ cells in 100 μl chemotaxis medium. Cells were allowed to migrate across the pores of the transwell inserts (pore size 5 μm) at 37°C and 5% CO_2_. After 4 h, transmigrated cells from a pool of three wells were harvested by centrifugation (5 min, 300 ×g), resuspended in 100 μl PBS containing 2 mM EDTA, stained with trypan blue and counted using a hemocytometer. The percentage of NK cells before and after transmigration was analyzed by flow cytometry. Briefly, cells were first incubated with anti-CD16/CD32 (2.4G2, BD Pharmingen) for 10 min on ice followed by incubation with anti-CD3ε (FITC, 145-2C11, BD Pharmingen), anti-NK1.1 (APC-Cy7, PK136, BioLegend), anti-NKp46 (BV450, 29A1.4, BD Bioscience) for 30 min on ice. After washing, analysis was performed on a BD LSRFortessa (BD Biosciences) flow cytometer. Data are expressed as percentage of input calculated by the following equation: migrated NK cell number/input NK cell number.

### Degranulation Assay

For the functional assessment of NK cells splenocytes were co-incubated with YAC-1 target cells at an effector:target ratio of 10:1 for a total incubation time of 6 h at 37°C and 5% CO_2_ in medium containing anti-CD107a antibody (BV421, 1D4B, BioLegend). After 1 h of the incubation, the co-culture was supplemented with 5 μg/ml monensin and brefeldin A (Sigma-Aldrich) to prevent receptor internalization and cytokine secretion, respectively. Subsequently, the cells were stained for the identification of NK cells (CD3^−^NK1.1^+^), perforin, granzyme B (GrB), and IFN-γ using the following antibodies: anti-CD3ε (BUV395, 17A2, BD Biosciences), anti-NK1.1 (APC, PK136, eBioscience), anti-perforin (PE, S16009B, BioLegend), anti-GrB (FITC, NGZB, eBioscience), and anti-IFN-γ (BV785, XMG1.2, BioLegend). For intracellular staining of perforin, GrB and IFN-γ, cells were permeabilized and fixed using the Cytofix/Cytoperm kit from BD according to the manufacturer's protocol. Briefly, cells were incubated in fixation buffer and subsequently stained with the antibodies diluted in permeabilization buffer.

### Statistics

All statistical analyses were performed using the GraphPad Prism Software v5 and v8 (GraphPad Software, Inc., La Jolla, USA) and *p* < 0.05 was considered as significant.

## Results

### Cytotoxic Capacity of NK Cells Primed During *In vivo Listeria monocytogenes* Infection Requires ADAP

**Figure 1:**
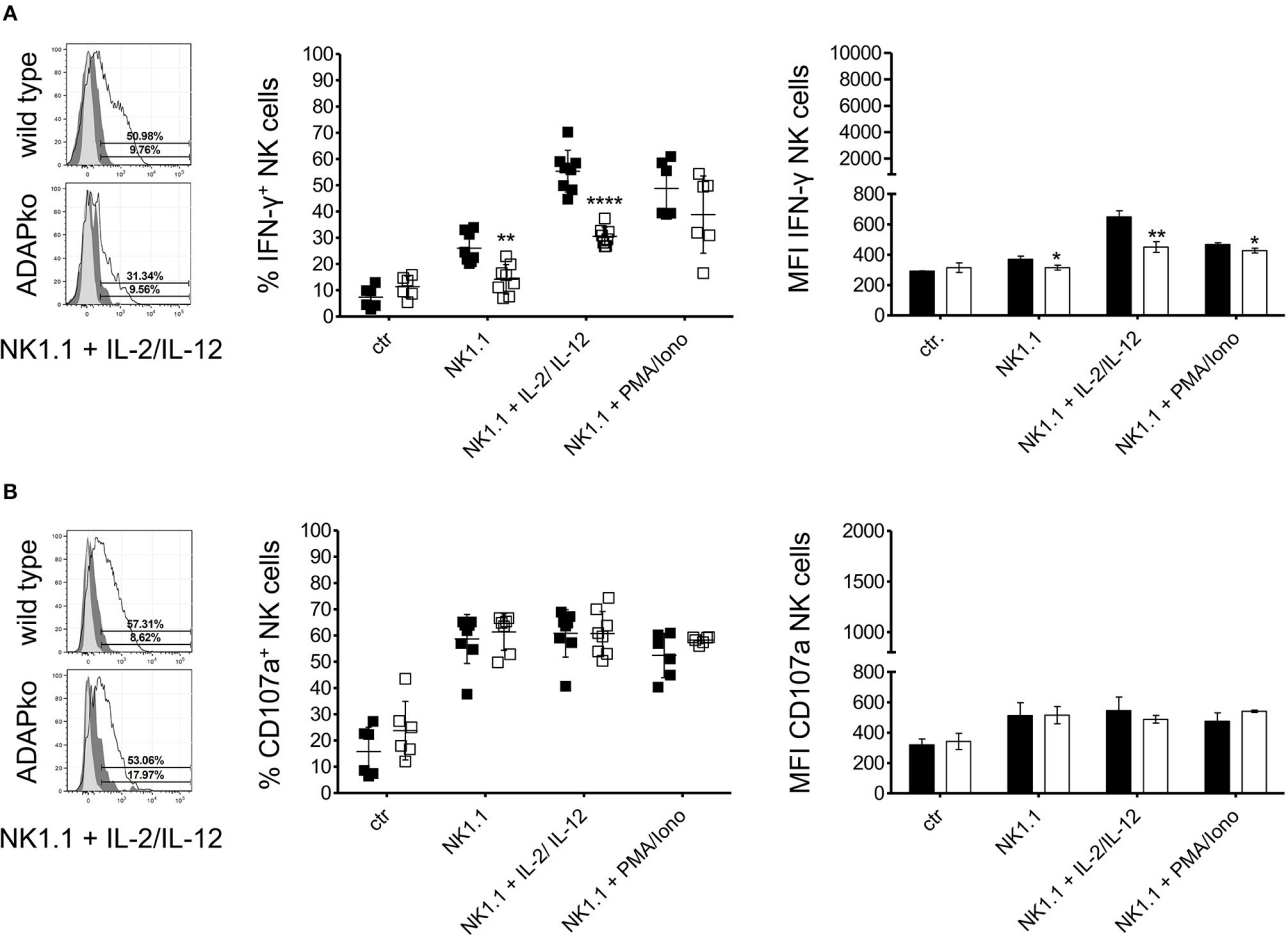
IFN-γ production in naïve NK cells depends on ADAP. Naïve NK cells from the spleen of wild type (black symbols) and ADAPko (open symbols) mice were either stimulated *in vitro* with anti-NK1.1 (plate bound) alone or in combination with IL-2/IL-12 or PMA/ionomycin for 4 h or were left unstimulated (ctr). Subsequently, frequency of **(A)** IFN-γ producing NK cells and **(B)** frequency of NK cells exhibiting CD107a surface expression as indicator for degranulation was analyzed by flow cytometry. Representative histograms (left panels) are shown for re-stimulation with anti-NK1.1 in combination with IL-2 and IL-12 (light gray: unstained and untreated; dark gray: stained and untreated; open: stained and treated). Mean ± SD of IFN-γ^+^ and CD107a^+^ CD3^−^CD49^+^ NK cells (middle panels) and mean fluorescence intensity (MFI, right panels) of IFN-γ in and CD107a on NK cells is shown with *n* = 6–8 individually analyzed mice per group out of three independent experiments. Groups were compared by unpaired two-tailed *t*-test with Welch's correction (*p < 0.05, **p < 0.01, ****p < 0.0001).

**Figure 2:**
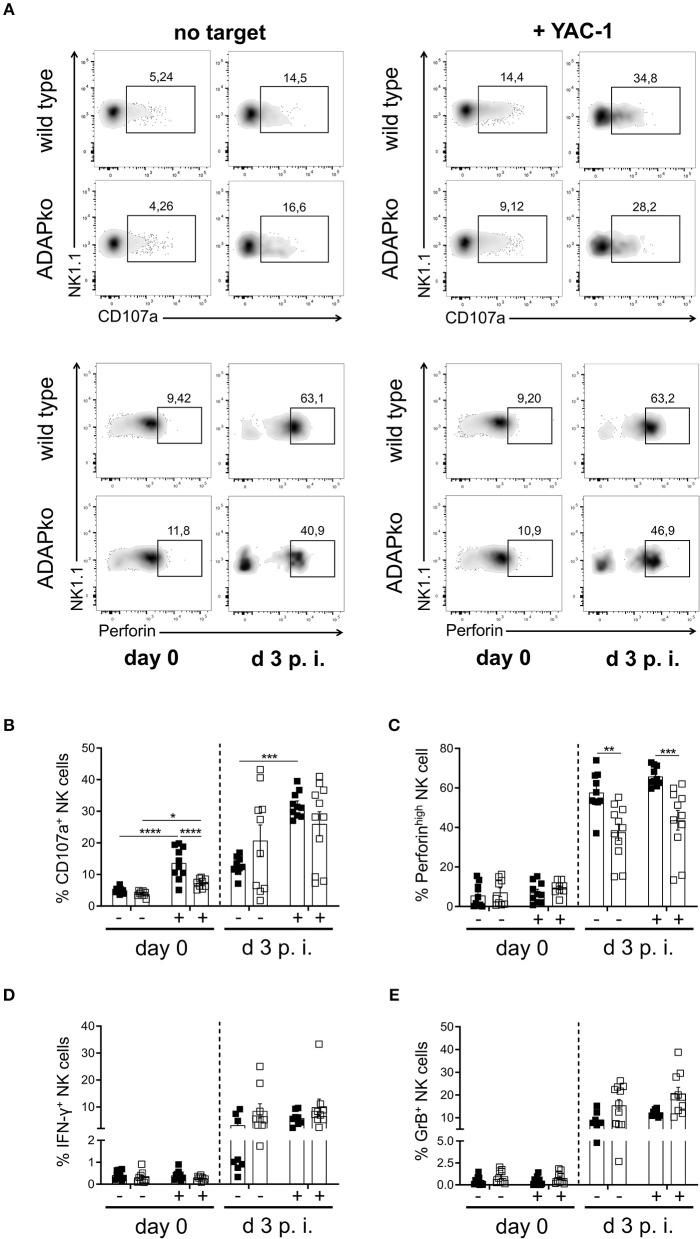
Degranulation capacity and perforin production by NK cells during *Lm* infection depend on ADAP. Wild type (black symbols) and ADAPko (open symbols) mice were either infected with 2.5 × 10^4^ CFU *Lm* or were left untreated (uninfected control, day 0) and were sacrificed at the indicated time points post infection. Splenocytes were isolated and incubated *in vitro* without targets (-) or with YAC-1 target cells (+). **(A)** Representative dot plots showing surface CD107a or intracellular perforin vs. NK1.1 expression on wild type and ADAPko splenocytes from naïve mice (day 0) as well as on day 3 post-*Lm* infection in the presence or absence of YAC-1 target cells. Frequency of **(B)** surface CD107a^+^, **(C)** perforin^high^, **(D)** IFN-γ^+^, and **(E)** granzyme B (GrB)^+^ CD3^−^NK1.1^+^ NK cells in spleen of uninfected mice (day 0) and day 3 post-*Lm* infection after *in vitro* co-incubation with YAC-1 target cells. Data are presented as mean ± SEM of *n* = 10 individual mice per group out of two independent experiments. Groups were compared by two-way ANOVA with Bonferroni correction for multiple hypothesis testing (*p < 0.05, **p < 0.01, ***p < 0.001, ****p < 0.0001).

To first confirm the role of ADAP in cytokine production by naïve NK cells, they were isolated from the spleen of naïve wild type and conventional ADAPko mice and were stimulated *in vitro* with anti-NK1.1 alone or in combination with IL-2/IL-12 or with PMA/ionomycin followed by flow cytometric analysis of IFN-γ production. As shown in Figure 1A, secretion of the effector cytokine IFN-γ by NK cells from ADAPko mice was significantly impaired compared to NK cells from ADAP sufficient mice. Importantly, wild type mice did not only exhibit higher frequencies of IFN-γ^+^ NK cells (% IFN-γ^+^ NK cells, Figure 1A, middle panel), but NK cells from wild type mice as well produced higher levels of IFN-γ (MFI IFN-γ NK cells, Figure 1A, right panel). We next analyzed whether, in addition to effector cytokine production, ADAP deficiency would also affect the cytotoxic capacity of naïve NK cells. To this end, splenic NK cells from naïve wild type and ADAPko mice were stimulated *in vitro* as described before followed by the quantification of surface expression of CD107a on NK cells as an indicator for preceeding NK cell degranulation (44). While under these experimental conditions ADAP deficiency significantly impaired IFN-γ production, it did not affect the degranulation capacity of naïve NK cells (Figure 1B) which is well in line with published data (12). We next extended our analyses to an experimental setting allowing NK cell priming within their natural environment, i.e., during *in vivo* infection, and to a more physiological NK cell *in vitro* stimulation set-up using YAC-1 target cells. To this end, wild type and conventional ADAPko mice were sublethally infected with *Lm*. Subsequently, NK cells were isolated from the spleen of wild type and conventional ADAPko mice on day 3 post *Lm* infection, together with NK cells from uninfected mice (day 0) serving as internal control. In contrast to our data obtained with antibody/cytokine stimulation, YAC-1-mediated stimulation of naïve NK cells uncovered a striking and ADAP-dependent degranulation defect as indicated by significantly reduced CD107a expression on ADAPko compared to wild type NK cells (Figures 2A,B). This phenotype became evident not only in YAC-1 stimulated naïve NK cells but was detectable as well in NK cells that were primed during *in vivo Lm* infection. While *in vitro* re-stimulation of NK cells from infected wild type animals with YAC-1 target cells stimulated a highly significant degranulation of these cells, YAC-1 cells did not induce further degranulation of *in vivo* primed ADAPko NK cells (Figures 2A,B). Consistent with their increased degranulation capacity *Lm* infection triggered NK cells to produce elevated levels of perforin and strikingly, this acquisition of NK cell effector function was significantly impaired in mice lacking ADAP as indicated by the reduced frequency of perforin^high^ ADAPko NK cells (Figures 2A,C). As for CD107a and perforin, there was a clear increase in the frequency of granzyme B (GrB) and IFN-γ producing NK cells on day 3 post-infection compared to uninfected controls, indicative for efficient *in vivo* priming, but no genotype-dependent differences were observed (Figures 2D,E).

To rule out the possibility that the striking differences observed for NK cell degranulation and perforin production was the consequence of inefficient NK cell priming in the ADAP-deficient host rather than an intrinsic effect of ADAP-deficiency in NK cells we compared serum concentrations of cytokines that have been described in the context of NK cell activation (26, 27, 45, 46). Serum cytokine levels on day 1 post-*Lm* infection were, with the exception of IL-1α, largely comparable in wild type and ADAPko mice. Interestingly, for several analytes known to be important for the stimulation of cytotoxicity in NK cells we even observed elevated serum concentrations in ADAPko mice on day 3 post-infection (Supplementary Figure 1A). Additionally performed quantitative gene expression analysis for the major NK cell activating cytokines IL-2, IL-12, IL-15, IL-18, and IL-21 in splenocytes from *Lm* infected mice revealed equal expression levels of these NK cell activating cytokines (Supplementary Figure 1B). Taken together, we show here that *Lm* infection in ADAP-deficient hosts induces a cytokine response largely comparable or even slightly stronger than in wild type animals and we thus conclude that the cytotoxic capacity of NK cells primed during *in vivo Lm* infection is intrinsically dependent on ADAP.

### ADAP-Deficiency Does Not Affect NK Cell Morphology, Intracellular Vesicle Distribution and the Overall Pattern of Protein Abundances in NK Cells During *Listeria monocytogenes* Infection

**Figure 3:**
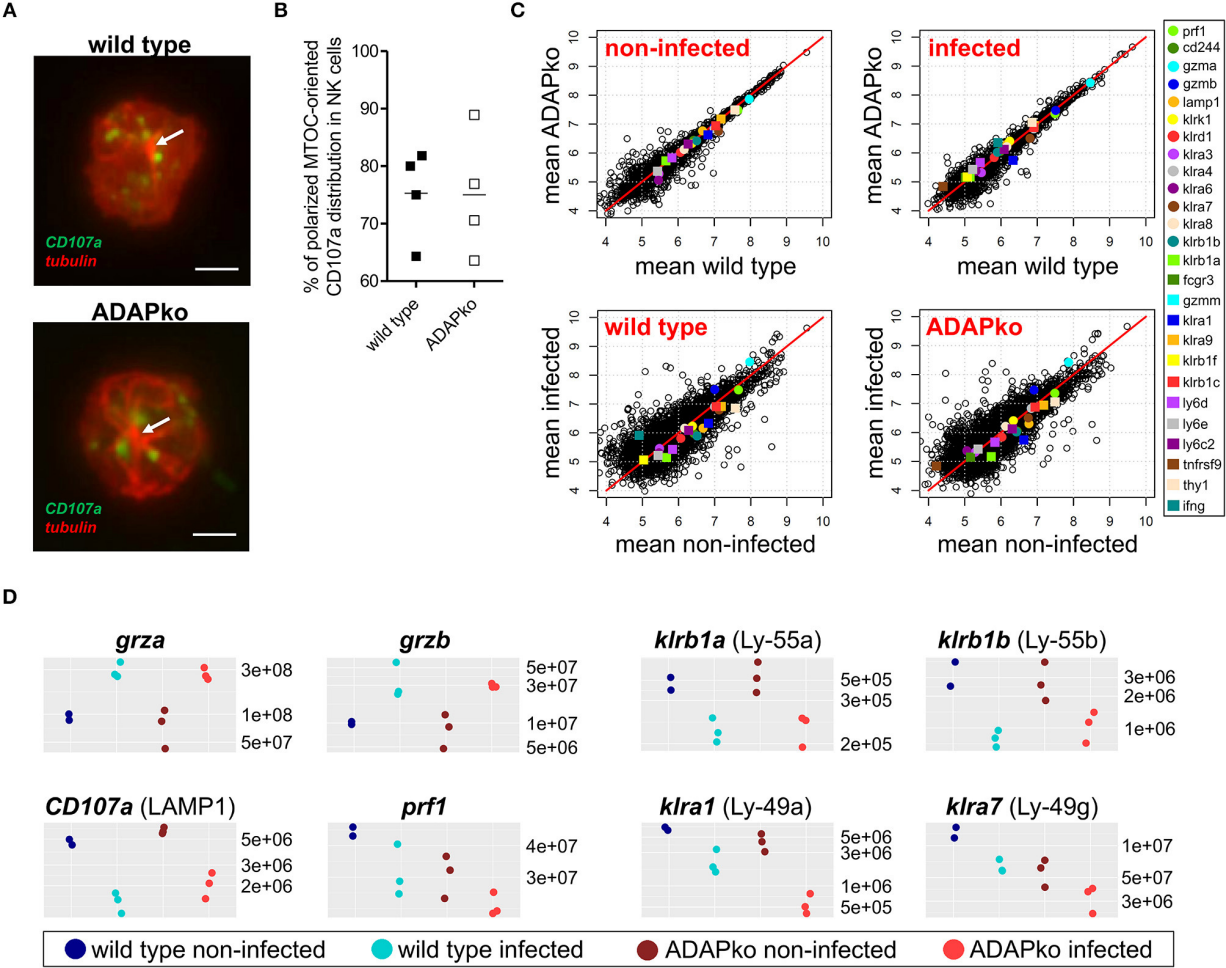
CD107a distribution and global NK cell protein composition are similar in wild type and ADAPko NK cells. Wild type and ADAPko mice were either infected with 2.5 × 10^4^ CFU *Lm* or were left untreated (non-infected control, day 0). Mice were sacrificed and NK1.1^+^ NK cells were isolated by flow cytometry and prepared for microscopy or mass spectrometry. **(A)** NK cells were seeded onto poly-L-lysine coated coverslips and stained for CD107a (green) and α-tubulin (red). Representative images of NK cells from *Lm* infected mice are given as stacked images. Scale bars are 2 μm. **(B)** Quantification of CD107a distribution toward the MTOC in NK cells from *Lm* infected mice (*n* = 4; up to 25 cells per animal were analyzed). **(C,D)** NK cells were lysed and analyzed by high-resolution mass spectrometry after tryptic digestion. **(C)** Scatter plots depicting mean log10 abundances (*n* = 3; for wild type day 0: *n* = 2) of all detected proteins (circles and squares) and selected NK cell proteins are marked in colors (color code given on the right panel). **(D)** Dot plots depicting absolute abundances of selected NK cell proteins for each individual mouse in all four conditions.

While we observed a clear impact of ADAP-deficiency on NK cell degranulation this effect became apparent only after YAC-1-mediated but not after antibody- and cytokine-mediated *in vitro* stimulation of NK cells (Figures 1, 2). Since ADAP deficiency in T cells prevents microtubule-organizing center (MTOC) translocation upon activation (47), we wondered whether ADAP-deficiency *per se* would affect NK cell morphology, microtubule network structures and distribution of vesicles, and whether such effect would become visible early on during *in vivo* infection. To analyze this, NK1.1^+^ NK cells were sorted from the spleen of *Lm* infected mice (4 mice for each genotype) 1 day post-infection and analyzed by microscopy without any further *in vitro* stimulation. As shown in Figure 3A, independent of the genotype NK cells exhibited a well-structured microtubule network with single microtubules originating from the MTOC (visualized with a white arrow in Figure 3A). Interestingly, counterstaining for CD107a identified CD107a^+^ vesicles (in green) distributed in the periphery of the MTOC, and again we did not observe differences in NK cells isolated from infected wild type or ADAPko mice (Figure 3A). Quantification of polarized CD107a distribution toward the MTOC in NK cells from *Lm* infected mice revealed indeed no differences. For both wild type and ADAPko mice, ~65 to ~90% of the NK cells showed MTOC-oriented CD107a distribution (Figure 3B). Similarly, NK cells isolated from naïve wild type and ADAPko mice showed no differences in CD107a distribution (data not shown). Thus, *ex vivo* analysis of NK cells at an early phase of *Lm* infection did not uncover obvious ADAP-dependent differences regarding cellular morphology and CD107a localization in vesicle-like structures. In line with this, phalloidin staining of cytoskeletal F-actin in naïve NK cells stimulated *in vitro* with CXCL12 did not reveal any significant difference in terms of actin re-organization between the genotypes as well (data not shown).

We further extended our molecular phenotyping and applied unbiased high-resolution mass spectrometry in order to identify potential ADAP-dependent alterations of NK cell proteomes in infected and non-infected mice. As for confocal microscopy, splenic NK cells from wild type and ADAPko mice were isolated on day 1 post *Lm* infection and analyzed without further stimulation in comparison with corresponding NK cells from naïve wild type and ADAPko mice using a label-free quantitative proteomics approach (LC-MS). We analyzed NK cells of six individual ADAPko and five individual wild type mice and quantified relative abundance of in total 4,131 proteins. In the individual samples, we identified 3,365–3,705 proteins with a normal distribution in protein abundances indicating the robustness of our analytical workflow (Supplementary Figure 2A). As expected, mass spectrometry identified ADAP exclusively in NK cells derived from wild type but not ADAPko mice (data not shown) underlining the accuracy of our analysis. Moreover, as a proof-of-concept, SKAP1, a protein that is co-regulated with ADAP expression (48), was undetectable in NK cells from infected ADAPko mice.

Computational cluster analysis of total NK cell proteomes clearly discriminated NK cells from infected and non-infected mice, but did not differentiate between NK cells from wild type and ADAPko mice (Supplementary Figure 2B). Likewise, comparison of protein intensities exhibited higher variation in NK cells from infected vs. non-infected mice (Figure 3C), underscoring that the infection-related *in vivo* priming of NK cells, but not the presence or absence of ADAP is decisive for the global changes observed in the proteome composition of NK cells. This held true for a number of prototypic NK cell proteins that were generally detectable at similar abundance in NK cells from non-infected wild type and ADAPko mice (Figure 3D). Among those the abundance of granzyme A and granzyme B increased upon *Lm* infection, while other proteins related to NK cell functions including CD107a, perforin and the Killer cell lectin-like receptor (KLR) subfamily members Ly-49a, Ly-49g, Ly-55a, and Ly-55b were detected with reduced abundance in NK cells from the infected host independent of the genotype (Figure 3D). In conclusion, unbiased *ex vivo* proteome profiling of splenic naïve NK cells and NK cells analyzed on day 1 post infection clearly revealed *in vivo* responsiveness of NK cells to *Lm* infection but did not uncover any obvious ADAP-dependent alterations in the effector molecule inventory involved in this process.

### NK Cell Migration, but Not *Listeria monocytogenes*-Induced IL-10 Production, Is Dependent on ADAP

**Figure 4:**
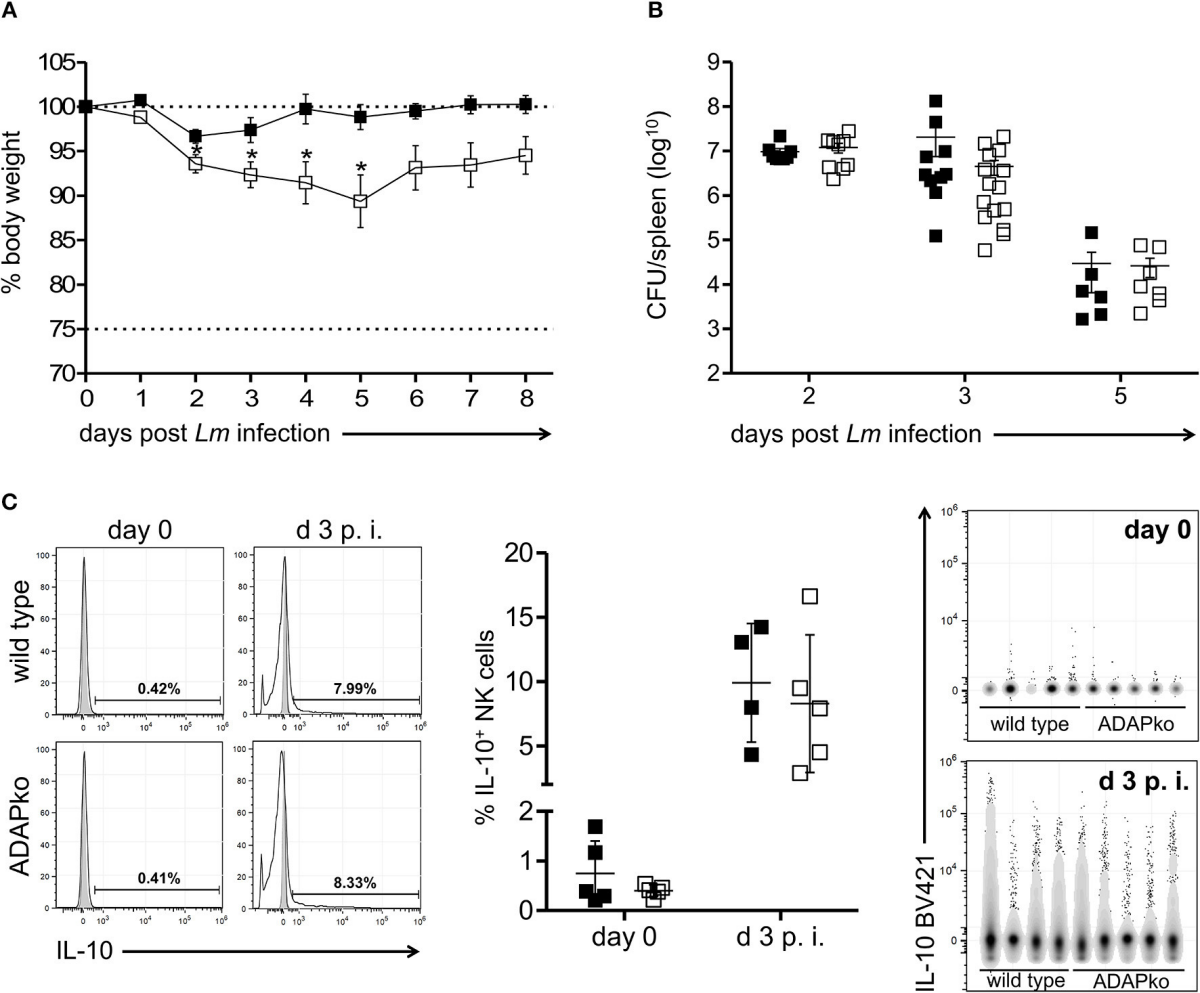
Enhanced susceptibility of conditional ADAPko mice to *Listeria monocytogenes* infection is not associated with altered IL-10 production by NK cells. ADAP^fl/fl^ × NKp46-Cre^het^ (open symbols) mice lacking ADAP specifically in NK cells and respective littermate controls (ADAP^wt/wt^ × NKp46-Cre^het^, black symbols) were either infected with **(A)** 5 × 10^4^ CFU *Lm*, **(B,C)** 2.5 × 10^4^ CFU *Lm* or were left untreated (uninfected control, day 0). **(A)** Body weight loss of ADAP^wt/wt^ × NKp46-Cre^het^ control mice and conditional ADAP^fl/fl^ × NKp46-Cre^het^ mice during the course of *Lm* infection**. (B)** CFU was quantified in the spleen as a measure for the bacterial load. Data are presented as mean ± SEM for *n* = 6–15 individually analyzed mice per group out of three independent experiments. **(C)** Representative histograms (left panels) are shown for hepatic CD3^−^NK1.1^+^NKp46^+^ IL-10 producing NK cells (light gray: unstained and untreated; dark gray: respective stained fluorescence minus one control; open: stained liver sample) for naïve NK cells (day 0, female mice) and day 3 post-*Lm* infection. Summary plots (middle panel) show percentage of IL-10 producing NK cells in liver. Data are presented as mean ± SD with *n* = 4–5 individual mice per group out of one experiment. Data were constrained to alive singlet NK cells and are shown in columns side-by-side in a concatenated qualitative dot plot (right panels) in which each column represents data of an individual mouse. Groups were compared by unpaired two-tailed *t*-test with Welch's correction (*p < 0.05).

We finally asked if and how ADAP-deficiency in NK cells would affect the overall course of *Lm* infection. To rule out any effect of ADAP-deficiency in immune cells other than NK cells we used conditional knock out mice lacking ADAP specifically in NK cells (ADAP^fl/fl^ × NKp46-Cre^het^). Strikingly, health monitoring during infection revealed enhanced severity of the disease as indicated by a significantly higher body weight loss in conditional ADAPko mice compared to control animals (Figure 4A). This was, however, not due to impaired antibacterial immunity since both genotypes controlled pathogen growth equally well (Figure 4B). Since IL-10 produced by NK cells has been recently shown to enhance susceptibility of mice to Listeria infection (34) we analyzed whether ADAP-deficiency would affect the capacity of NK cells to produce IL-10. While NK cells from uninfected mice did not produce relevant concentration of IL-10, IL-10 production was induced in NK cells by day 3 following *Lm* infection (Figure 4C). This was however genotype independent, thus largely excluding the possibility that enhanced morbidity of conditional ADAPko mice is due to enhanced IL-10 production by ADAP-deficient NK cells.

**Figure 5:**
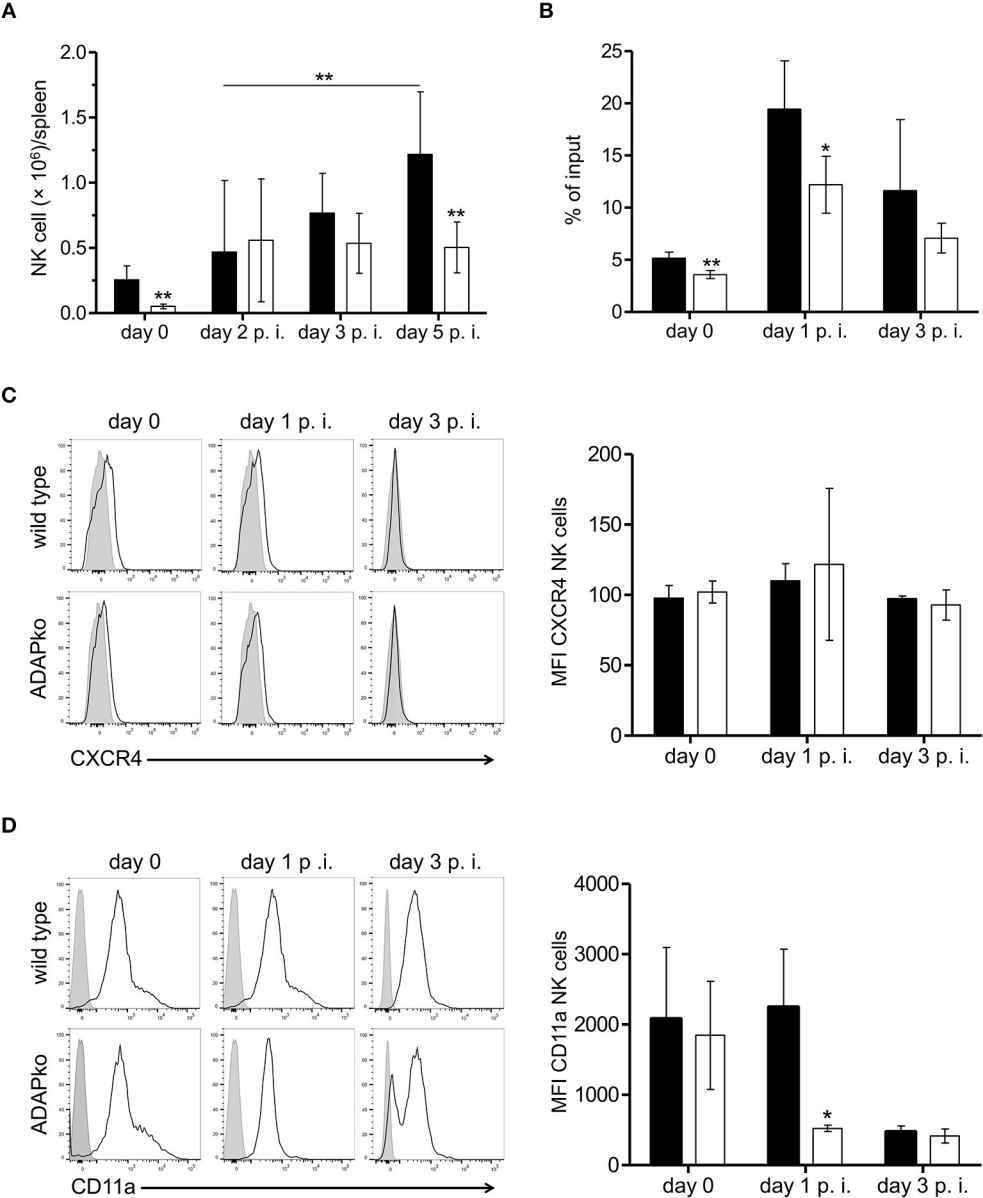
Reduced accumulation in the spleen and impaired migratory capacity of ADAP-deficient NK cells of *Lm* infected mice. ADAP^fl/fl^ × NKp46-Cre^het^ (open bars) mice lacking ADAP specifically in NK cells and respective littermate controls (ADAP^wt/wt^ × NKp46-Cre^het^, black bars) were either infected with 2.5 × 10^4^ CFU *Lm* or were left untreated (uninfected control, day 0). **(A)** ADAP^wt/wt^ × NKp46-Cre^het^ and conditional ADAP^fl/fl^ × NKp46-Cre^het^ mice were sacrificed at the indicated time points. Leukocytes were isolated from the spleen and NK cells were identified as CD3^−^NK1.1^+^CD49b^+^ cells. Absolute NK cell numbers were calculated from the NK cell frequencies assessed by flow cytometry referred to the absolute leukocyte numbers as well as the frequencies of live cells. Data are presented as mean ± SD for *n* = 4–15 mice per group out of three independent experiments. **(B)** Splenocytes were seeded in the upper transwell chamber that was placed in medium containing the chemokine CXCL12 (250 ng/ml). After 4 h cells were recovered from the lower chamber, counted and analyzed by flow cytometry to determine the percentage of migrated NK cells (% of input). Data are presented as mean ± SD for *n* = 4 mice per group from two independent experiments. **(C)** Level of CXCR4 and **(D)** CD11a surface expression on NK cells. Depicted are representative histograms (left panels; light gray: respective stained fluorescence minus one control FMO; open: stained) and mean fluorescence intensity (MFI, right panels) ± SD of **(C)** CXCR4 and **(D)** CD11a on NK1.1^+^ NK cells analyzed day 0 (female mice), day 1 and day 3 p.i. with *n* = 4–5 individually analyzed mice per group out of one to two experiments. Groups were compared by **(A–D)** unpaired two-tailed *t*-test with Welch's correction and additionally **(A)** by two-way ANOVA with Bonferroni correction for multiple hypothesis testing (*p < 0.05, **p < 0.01).

Interestingly, lack of ADAP in NK cells was associated with considerably lower NK cell numbers in the spleen of conditional ADAPko compared to wild type mice. Notably, this was evident already in naïve mice (Figure 5A). While in wild type animals the number of NK cells steadily increased until day 5 post-*Lm* infection, the number of ADAPko NK cells in the spleen rose to a far lesser extend and reached the plateau already by day 2 post-*Lm* infection (Figure 5A). Flow cytometric analysis of NK cell maturation markers revealed only slight though in part significant differences in the maturation of ADAP-sufficient and -deficient NK cells in the spleen of *Lm* infected mice during the course of infection. However, the frequency of NK cells that reached the different maturation stages (CD27^−^CD11b^−^ (DN) → CD27^+^CD11b^−^ → CD27^+^ CD11b^+^ (DP) → CD11b^+^CD27^−^) was the same by day 5 post-infection (Supplementary Figure 3) and as such impaired NK cell maturation was largely excluded as a reason for the pronounced ADAP-dependent differences in NK cell abundance in the spleen of *Lm* infected mice.

Since ADAP has been shown before to be involved in the migratory capacity of other immune cells (37, 49) and moreover protein network analyses of our proteome analyses identified three pathways potentially affected by ADAP-deficiency in NK cells to be related to cell migration (data not shown), we speculated that the observed phenotype might at least in part be the consequence of impaired migration of ADAPko NK cells to the spleen of *Lm* infected mice. To experimentally address this hypothesis we performed *in vitro* NK cell migration assays using splenocytes from naïve and *Lm* infected mice. Indeed and well in line with our expectation, NK cell migration toward a chemokine gradient was significantly impaired both in naïve as well as in ADAP-deficient NK cells pre-activated *in vivo* during *Lm* infection (Figure 5B). Of note, reduced *in vitro* migration of ADAPko NK cells toward the NK cell attracting chemokine CXCL12 was not the consequence of altered surface expression of the CXCL12 receptor CXCR4 as we observed similar CXCR4 expression levels on ADAP-deficient and –sufficient NK cells (Figure 5C). Since leukocyte entry into tissues is generally guided by the interaction of integrins with their respective ligands on endothelial cells we analyzed surface expression of selected integrins on splenic NK cells from *Lm* infected wild type and conditional ADAPko mice. While ADAP-deficiency did not affect expression levels of CD18, CD29, and CD11b on NK cells (data not shown), we observed a striking difference between ADAP-deficient and wild type NK cells with respect to CD11a surface expression on day 1 post *Lm* infection with significantly lower CD11a expression levels on NK cells lacking ADAP (Figure 5D). Together our data suggest that in addition to effector cytokine production and cytotoxic capacity ADAP is required for efficient migration of NK cells both *in vitro* and *in vivo* and that reduced NK cell cellularity in infected tissue of conditional ADAPko mice might be, at least in part, the consequence of reduced CD11a surface expression on ADAP-deficient NK cells.

## Discussion

We have studied for the first time ADAP-dependency of NK cell functions during *in vivo* infection. Our experimental approach is fundamentally different from previous studies as we studied the impact of ADAP on principal NK cell functions following their *in vivo* priming during infection, i.e., under physiological conditions involving a plethora of infection-induced cytokines as well as a complex network of NK cell-activating and inhibitory receptors. As such we feel that data from our *in vivo* study cannot directly be compared with the outcome from previous *in vitro* experiments but instead complement published knowledge and provides further clarification regarding the role of ADAP in infection-primed NK cells.

By extensively studying signaling events induced in NK cells by stimulation of the activating receptors CD137 or NKG2D, Rajasekaran et al. uncovered an ADAP-dependent signaling pathway exclusively responsible for the production of inflammatory cytokines but not for cytotoxicity (12). As an underlying mechanism for this striking dichotomy a unique interaction between Fyn and ADAP was discovered linking upstream signaling to the CBM module which, under the chosen experimental conditions, led to the production of IFN-γ and chemokines while not being responsible for cytotoxicity in murine and human NK cells (12). While applying antibody/cytokine-mediated *in vitro* stimulation of primed naïve NK cells confirmed these data (Figure 1), more physiological stimulation of naïve NK cells or infection-primed ADAPko NK cells with YAC-1 target cells uncovered impaired cytotoxicity of ADAP-deficient NK cells while their capacity to produce IFN-γ was not affected (Figure 2). Vivier et al. brought into consideration that the above mentioned study utilized IL-2-activated NK cells and that, given the plasticity of NK cells that is heavily dependent on their activation (50), it would be questionable if the findings by Rajasekaran et al. would generally apply to NK cells activated under different conditions (12). During Listeria infection various cytokines are induced that are known to permit NK cell activation (51) and indeed most of the known NK cell-activating cytokines were detectable in sera of *Lm* infected wild type and ADAPko mice (Supplementary Figure 1). Importantly, ADAPko mice did not exhibit an overall defect in cytokine response to *Lm* infection (Supplementary Figure 1). Thus, we can largely exclude that impaired effector function in ADAPko NK cells is due to impaired priming in the ADAP-deficient host but is rather an inherent effect due to ADAP-deficiency in NK cells. Apart from the proteome analysis (Figure 3) we did not further analyze the influence of *Lm* infection on the expression of activating/inhibitory receptors on NK cells and the expression of the corresponding ligands on target cells, respectively. Anyway, we hypothesize that during infection the combination of cytokines and/or receptor-ligand pairs is ideal to prime NK cells for both, cytokine production and cytotoxicity and that under these optimized priming conditions the separation of the ADAP-dependent and -independent signaling pathways in NK cells is abrogated. Future studies are needed to clarify this aspect on the molecular level.

ADAPko NK cells exhibit reduced cytotoxic capacity (Figure 2). This phenotype was further confirmed by proteomic profiling that on the one hand revealed an overall decrease of intracellular perforin and CD107a upon infection suggesting the release of perforin from CD107a^+^ granules following *in vivo* priming. Moreover, given that the applied LC-MS/MS analysis is particularly suitable for the detection of intracellular proteins, the observation of a slightly more pronounced decrease in CD107a protein abundance in ADAP-sufficient compared to ADAP-deficient NK cells during infection as well-hints at decreased cytolytic activity of ADAPko NK cells (Figure 3D).

Remarkably, independent of the genotype infection-priming of NK cells resulted in an overall decreased abundance of virtually all detectable prototypic NK cell proteins with the exception of granzyme A and granzyme B that were even found at higher abundances (Figures 3C,D). While at a first glance it might be counter-intuitive that priming of NK cells would result in increased granzyme A and granzyme B levels but at the same time decreased abundance of CD107a and perforin, several mechanisms could explain this finding. Already in previous studies we observed co-localization of CD107a with perforin but not with granzymes (Heyner et al., unpublished) and it has been shown that dependent on the activating signal used to stimulate NK cell function different cytotoxic vesicles are detectable within the cell. For instance, engagement of NKG2D or 2B4 on NK cells induces the co-localization of perforin with Rab27a^+^ but not Munc13-4^+^ vesicles. In direct contrast, antibody-dependent CD16 activation induces perforin localization to Munc13-4^+^ vesicles but not to Rab27a^+^ ones (52). Moreover, Munc13-4 and Rab27a have been described to be involved in granzyme B polarization to the immunological synapse (53). Thus, the observed differences regarding granzyme A/granzyme B vs. CD107a/perforin abundances might be a result of complex ligand/receptor binding events induced early on during infection leading to discrete maturation of lytic granules in NK cells. On the other hand, we cannot exclude that newly synthesized proteins, i.e., induced gene expression upon infection might also explain the differences of cytotoxic protein abundances. In this context, Fehniger et al. have shown that, well in line with our proteome data (Figure 3D), resting NK cells contain high levels of granzyme A but little granzyme B and perforin (54). Interestingly, they uncovered a clear discrepancy in terms of protein content *vs*. mRNA content, with mRNA being detectable in naïve NK cells at high abundance for all three genes. While naïve NK cells were *per se* granzyme A^+^, stimulation with IL-15 increased the frequency of granzyme B and perforin expressing NK cells. Only in case of granzyme B this was associated with induced mRNA expression (54). While these data do not explain the inverse pattern of granzyme A/granzyme B and perforin in infection-primed NK cells they provide evidence for fundamental differences regarding the regulation of transcription and translation for these cytotoxic mediators in NK cells. Still, both granzyme B and perforin were shown to be required for efficient cytotoxicity *in vitro* and *in vivo* (54) and we show here that the abundance of one of them, i.e., perforin seems to be diminished in NK cells lacking ADAP (Figure 2C).

Interestingly, ADAP-deficiency in NK cells was associated with a more severe course of *Lm* infection (Figure 4A) which was not due to impaired antibacterial immunity (Figure 4B). Activated NK cells respond to *Lm* infection (19) in a complex process that requires crosstalk with macrophages, neutrophils and dendritic cells (35, 36). With this, NK cells are able to shape the immune response *via* pro-inflammatory cytokine and chemokine stimulation as well as due to cell-to-cell contacts (55, 56). Enhanced disease severity might be indicative for a critical role of ADAP in NK cells in the overall innate immune response induced during *Lm* infection. Apart from the role of ADAP in NK cells during listeriosis it is however even not clear whether NK cells exhibit a beneficial or detrimental role in immunity to *Lm*. An early study by Teixeira and Kaufmann revealed improved pathogen control in mice lacking NK cells (57). The deleterious role of NK cells in listeriosis was confirmed later by Viegas et al. demonstrating that although NK cells are not required for pathogen elimination *Lm* infected NK cell-deficient mice showed improved survival (58). Data from another study however implied that NK cells may rather be protective during *Lm* infection (51). By secretion of the pro-inflammatory cytokines IL-12, TNF-α, IL-1β, and IL-18 by *Lm* activated dendritic cells, NK cells are primed to produce IFN-γ. IFN-γ plays a pivotal role in innate and adaptive immunity to intracellular bacteria and indeed mice lacking the IFN-γ receptor are highly susceptible to *Lm* infection (59). Despite comparable IFN-γ production in infection-primed wild type and ADAPko NK cells (Figure 2D) IFN-γ serum concentration was increased by day 3 p.i. in ADAPko mice (Supplementary Figure 1). However, since in conventional ADAPko mice also immune cells other than NK cells are deficient for ADAP, we assume that IFN-γ produced by e.g., T cells, NKT cells, macrophages, B cells, or dendritic cells accounts for the observed difference. This is supported by our finding that in conditional ADAPko mice lacking ADAP exclusively in NK cells, IFN-γ serum concentration markedly increases by day 2 post-*Lm* infection, while we did not observe significant differences in infection-induced IFN-γ levels between wild type and conditional ADAPko mice (data not shown). It has recently been shown that infection-induced NK cell activation increases susceptibility to *Lm* infection independent from IFN-γ production by NK cells (34). As an underlying mechanism for the detrimental role of NK cells in listeriosis the authors uncovered that NK cells responding to *Lm* infection acquire the ability to produce the immunosuppressive cytokine IL-10. In a follow-up study the same group identified that licensing of IL-10 production in NK cells requires IL-18 released by *Lm* infected Bat3^+^ DCs (60). We did not observe any effect of ADAP-deficiency in NK cells on their capacity to produce IL-10 (Figure 4C). Combined with the finding, that neither the concentration of the immunosuppressive IL-10, nor the NK cell licensing cytokine IL-18 differed in sera of *Lm* infected wild type and ADAPko mice (Supplementary Figure 1 and data not shown for conditional ADAPko mice), we exclude the possibility that IL-10 produced by NK cells would account for the enhanced morbidity of conditional ADAPko mice to *Lm* infection.

Within the early phase of *Lm* infection phagocytes are critically involved in immune containment of the pathogen (61). Infiltrating monocytes and especially neutrophils attracted to the site of infection may cause adverse side effects thus contributing to immunopathology (62, 63). Since infection-primed ADAPko NK cells exhibited impaired production of the phagocyte attracting chemokines CCL3, CCL4, and CCL5 (Supplementary Figure 4) we compared the number of monocytes and neutrophils in the spleen of *Lm* infected animals. However, no differences in the absolute numbers of monocytes and neutrophils were detectable (data not shown). Macrophages and neutrophils are considered the major source for IL-12 during listeriosis (64, 65). Notably, next to comparable numbers of these cells in the spleen of *Lm* infected wild type and conditional ADAPko mice we also did not find quantitative differences in serum IL-12 levels (data not shown) as a potential indicator for differential activation of phagocytes in mice lacking ADAP in NK cell. Taken together, enhanced body weight loss of infected conditional ADAPko mice most likely cannot be attributed to enhanced immunopathology exerted by phagocytes. However, the molecular/cellular mechanism by which ADAP-deficiency in NK cells promote disease severity during *Lm* infection remains elusive.

To the best of our knowledge, no data are available regarding the migration of ADAP-deficient NK cells. Utilizing an *in vitro* transwell system we could show for the first time that CXCL12-induced migration of ADAP-deficient NK cells is reduced compared to ADAP-sufficient NK cells. During infection, NK cells are recruited to the sites of inflammation in a chemokine-dependent manner. This is well-reflected by the observed increased migratory activity of *Lm* infection-primed compared to naïve NK cells (Figure 5B). CXCL12 is an important chemokine not only for T cells, but also for the chemo-attraction of NK cells (66). Since *Lm* infection in mice represents a complex disease model with different leukocyte subsets being recruited and activated to produce pro-inflammatory cytokines and chemokines during early innate immune activation, *in vitro* testing of CXCL12-induced migration of NK cells only insufficiently reflects the *in vivo* situation. Nevertheless, impaired migratory activity of ADAP-deficient NK cells might at least in part explain the reduced numbers of NK cells in the spleen of conditional ADAPko mice early after *Lm* infection (Figure 5A). In T cells, the inside-out signaling pathways leading to integrin activation after chemokine receptor stimulation are well-described (4) and we have recently shown that migration of both, CD4^+^ and CD8^+^ T cells, depends on ADAP (6). For NK cells, it is known that integrin-dependent activation is essential for migration and for cytotoxicity (67). Therefore, it seems likely that similar signaling complexes are formed in T cells and NK cells. Of note, compared to wild type NK cells, CD11a surface expression on infection-primed ADAPko NK cells was markedly reduced on day 1 post-infection (Figure 5D). Together with CD18, CD11a forms the heterodimeric adhesion molecule LFA-1 which upon interaction with its ligand ICAM on endothelial cells promotes entry of leukocytes from the bloodstream into tissues. In fact, an early study by Allavena et al. has shown that LFA-1 is crucial for NK cell adhesion to and migration through the vascular endothelium (68). Importantly, at least in T cells the intracellular domain of CD11a is directly linked to the signaling complex involving ADAP (69, 70). Thus, it is tempting to speculate that lack of ADAP in NK cells in NK cells and the associated reduced surface expression of CD11a on NK cells 1 day post *Lm* infection is mechanistically linked to the reduced accumulation of NK cells in the infected spleen of conditional ADAPko mice (Figure 5A). However, since differential CD11a expression on ADAPko vs. wild type NK cells was only transient and genotype-dependent differences in CD11a surface expression were lost by day 3 post-infection, most probably additional molecular factors will contribute to the observed phenotype. Yet, further studies are needed to dissect in more detail the molecular mechanism underlying reduced migratory activity of naïve and infection-primed ADAP-deficient NK cells.

## Data Availability Statement

The raw data supporting the conclusions of this article will be made available by the authors, without undue reservation, to any qualified researcher.

## Ethics Statement

The animal study was reviewed and approved by Niedersächsisches Landesamt für Verbraucherschutz und Lebensmittelsicherheit, Germany and Landesamt für Verbraucherschutz, Sachsen-Anhalt, Germany.

## Author Contributions

MB designed and performed experiments and wrote the manuscript. ST, PR, MHa, MHe, MV, and GP designed and performed experiments. FK performed data mining and statistical analysis for proteome data. AJ contributed conception to the analyses. LJ contributed conception to the analysis, data interpretation, and provided research material. CG provided research material. BS, AR, and DB designed and supervised the research and wrote the manuscript. All authors contributed to manuscript revision, read, and approved the submitted version.

### Conflict of Interest

The authors declare that the research was conducted in the absence of any commercial or financial relationships that could be construed as a potential conflict of interest.
