# Supplementary figures and images for: ADAP Promotes Degranulation and Migration of NK Cells Primed During in vivo Listeria monocytogenes Infection in Mice
Source: Front Immunol. 2020 Jan 22;10:3144. doi: 10.3389/fimmu.2019.03144 (PMC6987423; doi:10.3389/fimmu.2019.03144)

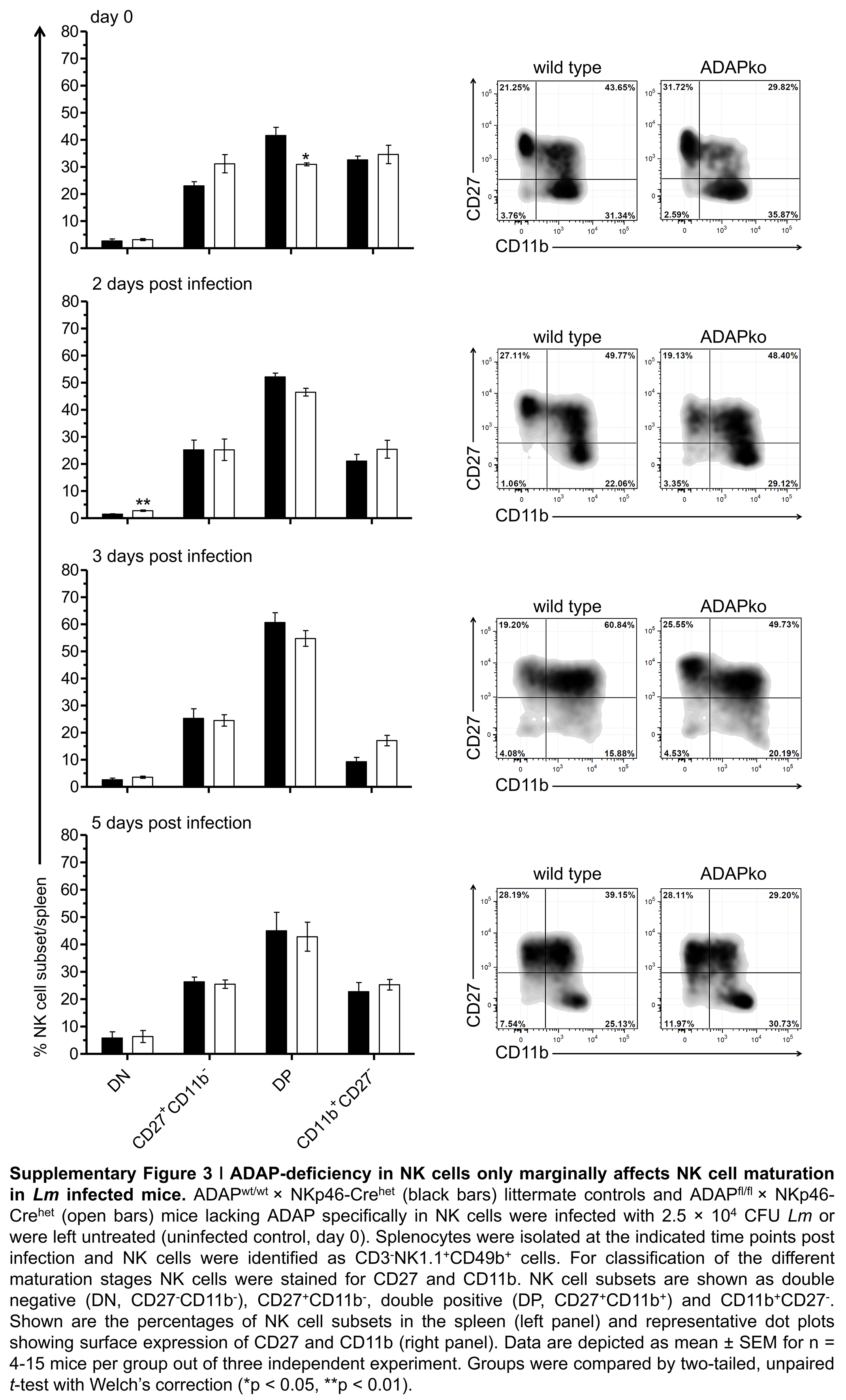

Supplement: Supplementary file 3 [file Image_3.TIF]

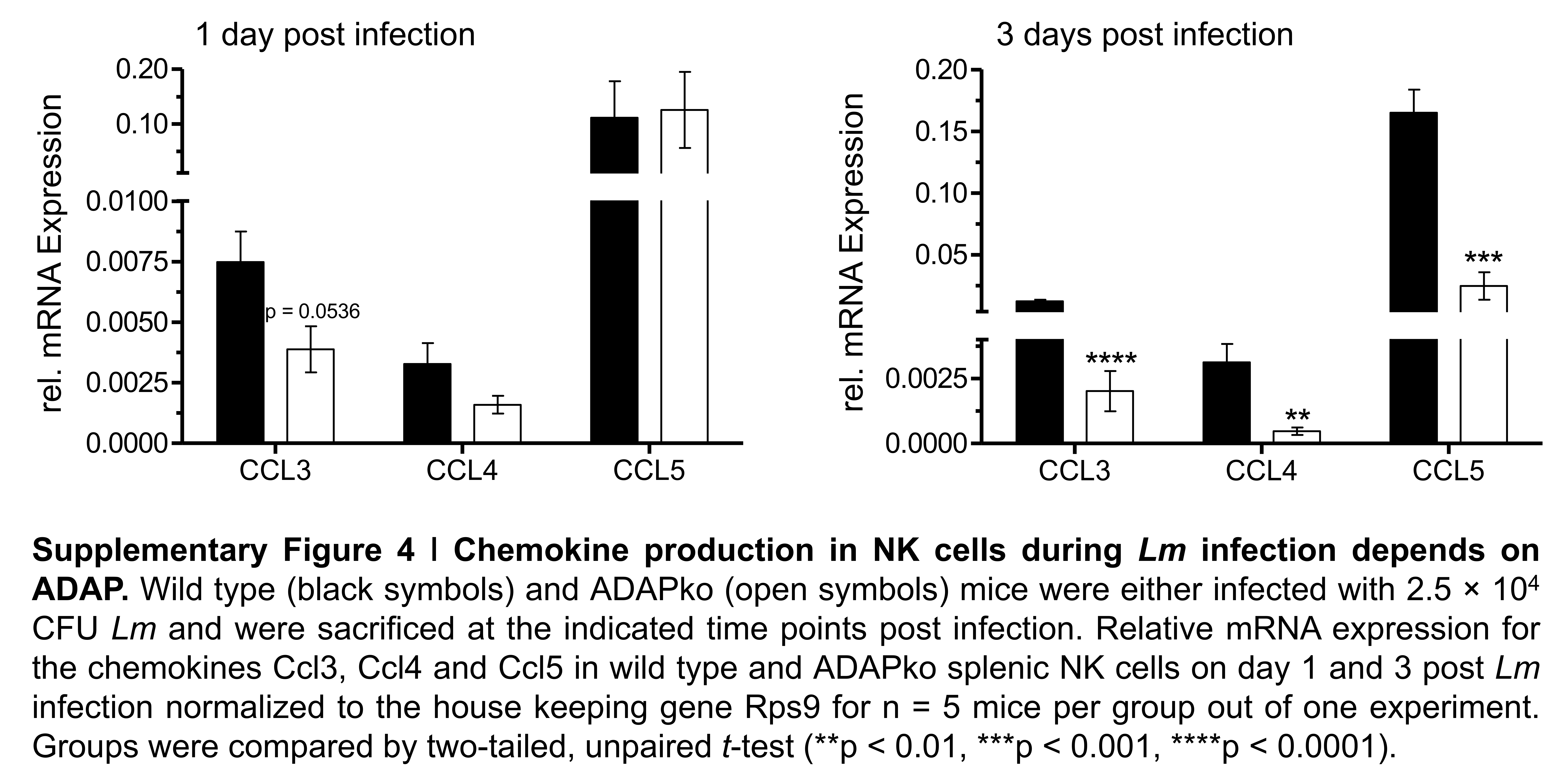

Supplement: Supplementary file 4 [file Image_4.TIF]
